# Acute inflammatory reaction during anti-angiogenesis therapy combined with immunotherapy as a possible indicator of the therapeutic effect: Three case reports and literature review

**DOI:** 10.3389/fonc.2023.1072480

**Published:** 2023-04-14

**Authors:** Yihui Lei, Li Lin, Shuyu Cheng, Qiming Shao, Chenchun Ding, Renjie Zuo, Weiping Chen, Quan Liao, Guoyan Liu

**Affiliations:** ^1^ The School of Clinical Medical, Fujian Medical University, Fuzhou, Fujian, China; ^2^ Department of Gastrointestinal Surgery, Zhongshan Hospital of Xiamen University, School of Medicine, Xiamen University, Xiamen, China; ^3^ Institute of Gastrointestinal Oncology, Medical College of Xiamen University, Xiamen, Fujian, China

**Keywords:** apatinib, camrelizumab, Tas-102, gastroenteric tumor, gastric cancer, colorectal cancer, acute inflammation

## Abstract

The posterior line treatment of unresectable advanced or metastatic gastrointestinal (GI) tumors has always been a challenging point. In particular, for patients with microsatellite stable (MSS)/mismatch repair proficient (pMMR) 0GI tumors, the difficulty of treatment is exacerbated due to their insensitivity to immune drugs. Accordingly, finding a new comprehensive therapy to improve the treatment effect is urgent. In this study, we report the treatment histories of three patients with MSS/pMMR GI tumors who achieved satisfactory effects by using a comprehensive treatment regimen of apatinib combined with camrelizumab and TAS-102 after the failure of first- or second-line regimens. The specific contents of the treatment plan were as follows: apatinib (500 mg/d) was administered orally for 10 days, followed by camrelizumab (200 mg, ivgtt, day 1, 14 days/cycle) and TAS-102 (20 mg, oral, days 1–21, 28 days/cycle). Apatinib (500 mg/d) was maintained during treatment. Subsequently, we discuss the possible mechanism of this combination and review the relevant literature, and introduce clinical trials on anti-angiogenesis therapy combined with immunotherapy.

## 1 Introduction

Gastrointestinal (GI) cancer is one of the most common cancers worldwide. From a global point of view, the incidence rate of GI tumors is all in the anterior position of tumors. Specifically, the incidence rate of colorectal cancer (CRC) accounts for about 10% of the total cases, ranking third in the world (1), whereas the incidence rate of gastric cancer (GC) accounts for 5.6% of the total cases, ranking fifth in the world (1). Recent advancements in the understanding of molecular biology and pathophysiology of gastroenteric cancer have increased the treatment option for advanced GI tumors. Treatments include extensive surgery, radio-frequency ablation, transcatheter arterial chemoembolization, stereotactic body radiation therapy, palliative chemotherapy, targeted therapy, and immunotherapy (2). These new treatments have significantly improved the overall survival (OS) rate of patients with advanced GC or CRC (3–7). For patients with advanced GI tumors suitable for surgery, resection is still the best way to achieve long-term survival. However, the prognosis of advanced GI tumors remains poor because of the high recurrence rate. According to previous studies, for patients undergoing radical resection of CRC, the postoperative recurrence rate or metastasis rate can reach 15.2%–25.7% (8–10). For patients with GC undergoing radical resection, about 38.8%–58.9% of patients with GC will still have postoperative recurrence or metastasis (11–13). This type of patients with postoperative recurrence or metastasis has always been difficult to treat. In recent years, various new immune drugs have emerged. Immune drugs based on programmed cell death protein-1 (PD-1)/programmed death-ligand 1 (PD-L1) blockade provide a new choice for the treatment of patients with postoperative recurrence or metastasis of GI tumors. However, most patients do not respond to PD-1/PD-L1 blockade, and some responders also develop acquired drug resistance after the initial response (14). Despite this, multiple studies have indicated that combining the treatment of anti-angiogenesis agents and PD-1/PD-L1 antibodies achieves synergistic effects on different types of cancer (15, 16), bringing hope to patients with advanced cancer. In the present case report, we discuss the treatment of three patients with microsatellite stable (MSS) advanced GI tumors. After the failure of the first- and second-line chemotherapy regimen, they were given the combined treatment regimen of apatinib plus camrelizumab and TAS-102, ultimately achieving satisfactory treatment results.

## 2 Case representation

### 2.1 Patient 1

**Figure 1:**
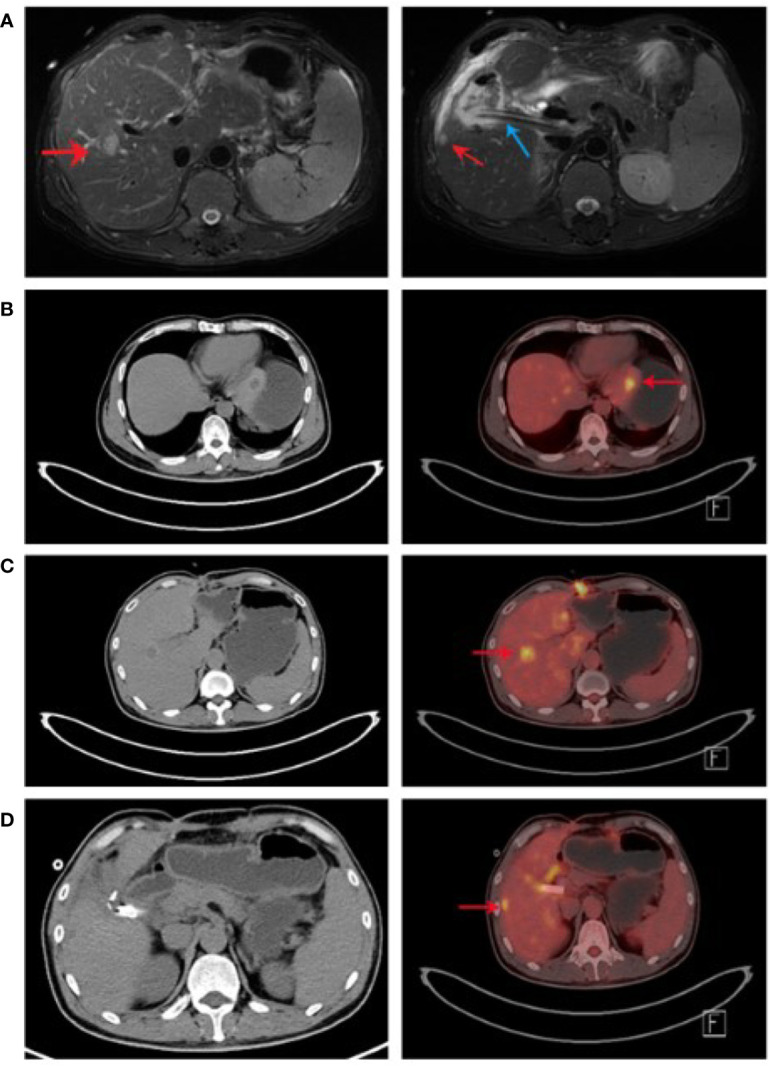
Re-examination of liver by MRI and PET-CT. **(A)** After the operation, liver MRI showed that the liver structure changed and metastatic nodules formed. The structure of the operation area became disordered, as shown in the shadow of the drainage tube (indicated by the blue arrow). The local signal was chaotic, and the enhancement was obvious. The remnant liverMRI of the liver had three round nodules. The larger one located in the left liver was about 1.8 cm × 1.4 cm (indicated by the red arrow in the left picture). A smaller one located in the right lobe was about 0.89 cm in diameter (indicated by the red arrow in the right picture). **(B-D)** PET-CT scan showed low-density nodules with sizes of 1.7 cm × 1.0 cm **(B)**, 0.8 cm × 0.8 cm **(C)**, and 1.5 cm × 1.4 cm **(D)**. The nodules are indicated by red arrows.

**Figure 2:**
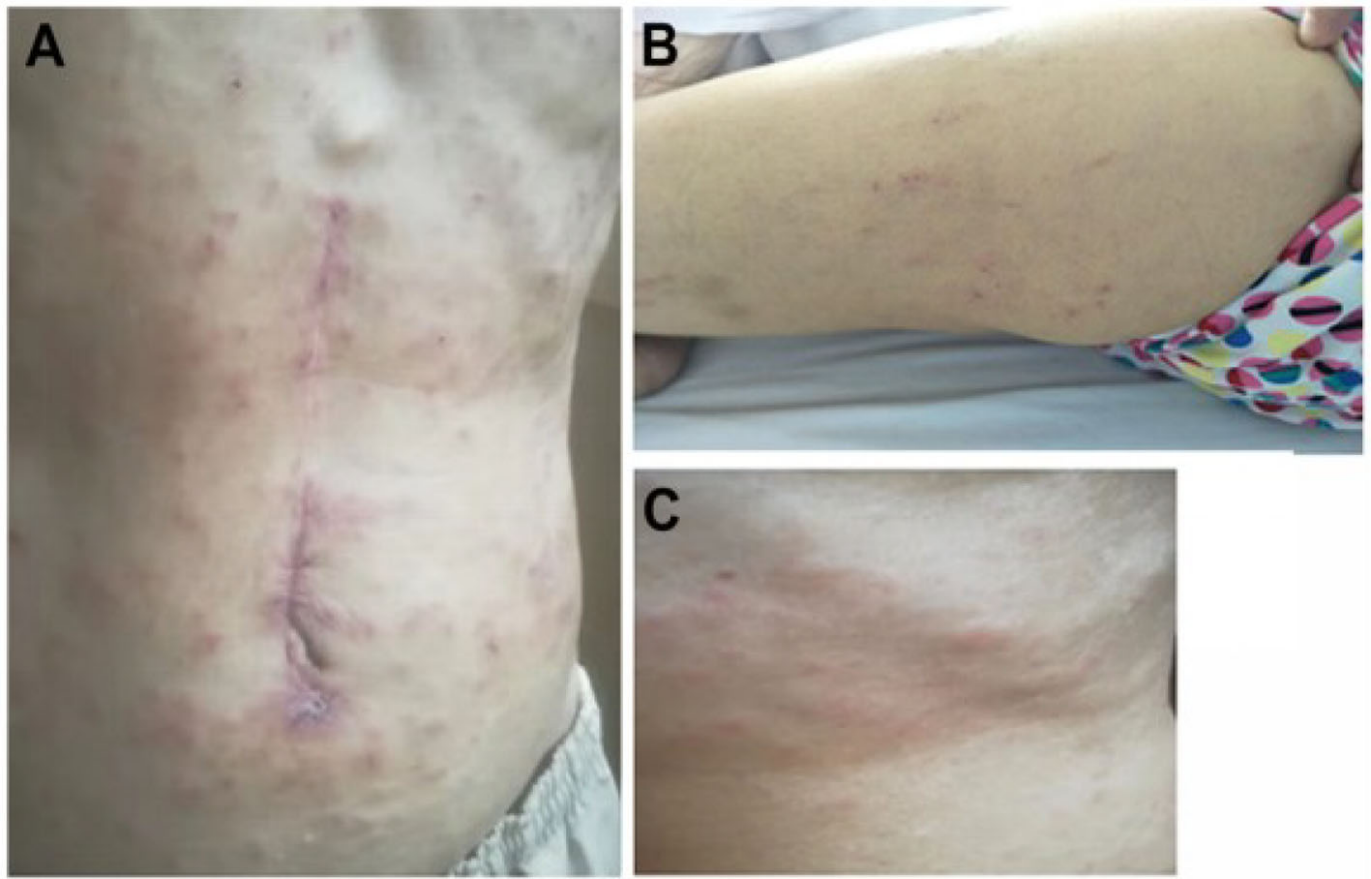
Skin rash were observed during the first cycle of combinative treatment. Skin rashes were observed in the front abdomen **(A)**, calf **(B)**, and neck **(C)**.

**Figure 3:**
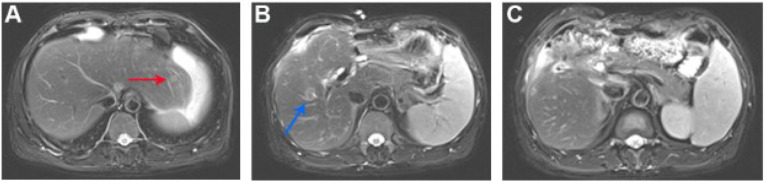
After five cycles of combinative treatment, MRI of the patient liver showed that liver metastasis sites diminished or disappeared. **(A)** The metastatic nodule in the left outer upper segment of the liver diminished (the red arrow showed the position of the previous nodule; **Figure 3A**); **(B)** The metastatic nodule in the center of the right upper liver diminished (the blue arrow showed the position of the previous nodule; **Figure 3B**); **(C)** The metastatic nodules under the capsule disappeared (**Figure 3C**).

A 47-year-old man was first diagnosed with transverse colon cancer with simultaneous liver metastasis in June 2018. On July 22nd, 2018, the patient received a radical resection of transverse colon cancer, liver section 5 and 6 segmental, and a cholecystectomy. Postoperative pathological analysis showed a diagnosis of moderately differentiated adenocarcinoma, consistent with the preoperative diagnosis, and the pathological staging was pT4aN2aM1a, IVA. After surgery, the patient received a 500 mg/day apatinib single-drug maintenance treatment. On September 27th, 2018, the re-examination of the patient’s liver by magnetic resonance imaging (MRI) revealed multiple metastases of the remnant liver (**Figure 1A**). A capecitabine and oxaliplatin (XELOX) 21-day treatment was then applied for two rounds. Four months after the surgery, re-examination by positron emission tomography (PET)–computed tomography (CT) scan showed low-density nodules in the remnant liver (**Figures 1B–D**). After multidisciplinary treatment (MDT) discussion, a combined treatment regimen was performed on 2018/12/2. In particular, apatinib (500 mg/d) was administered orally for 10 days, followed by camrelizumab (200 mg, ivgtt, day 1, 14 days/cycle) and TAS-102 (20 mg, oral, days 1–21, 28 days/cycle). Apatinib (500 mg/d) was maintained during treatment. After five times of combined treatment regimen, the carcinoembryonic antigen (CEA) level decreased from 188.7 ng/ml to 17.1 ng/ml. During the first cycle of treatment, the patient showed obvious systemic acute inflammatory reactions such as skin rash and oral ulcer (**Figure 2**). On 2019/3/25, MRI of the patient’s liver showed that the shape of the remnant liver was similar to that of the former one, and most of the primary metastases shrunk or disappeared (**Figures 3A–C**).

### 2.2 Patient 2

**Figure 4:**
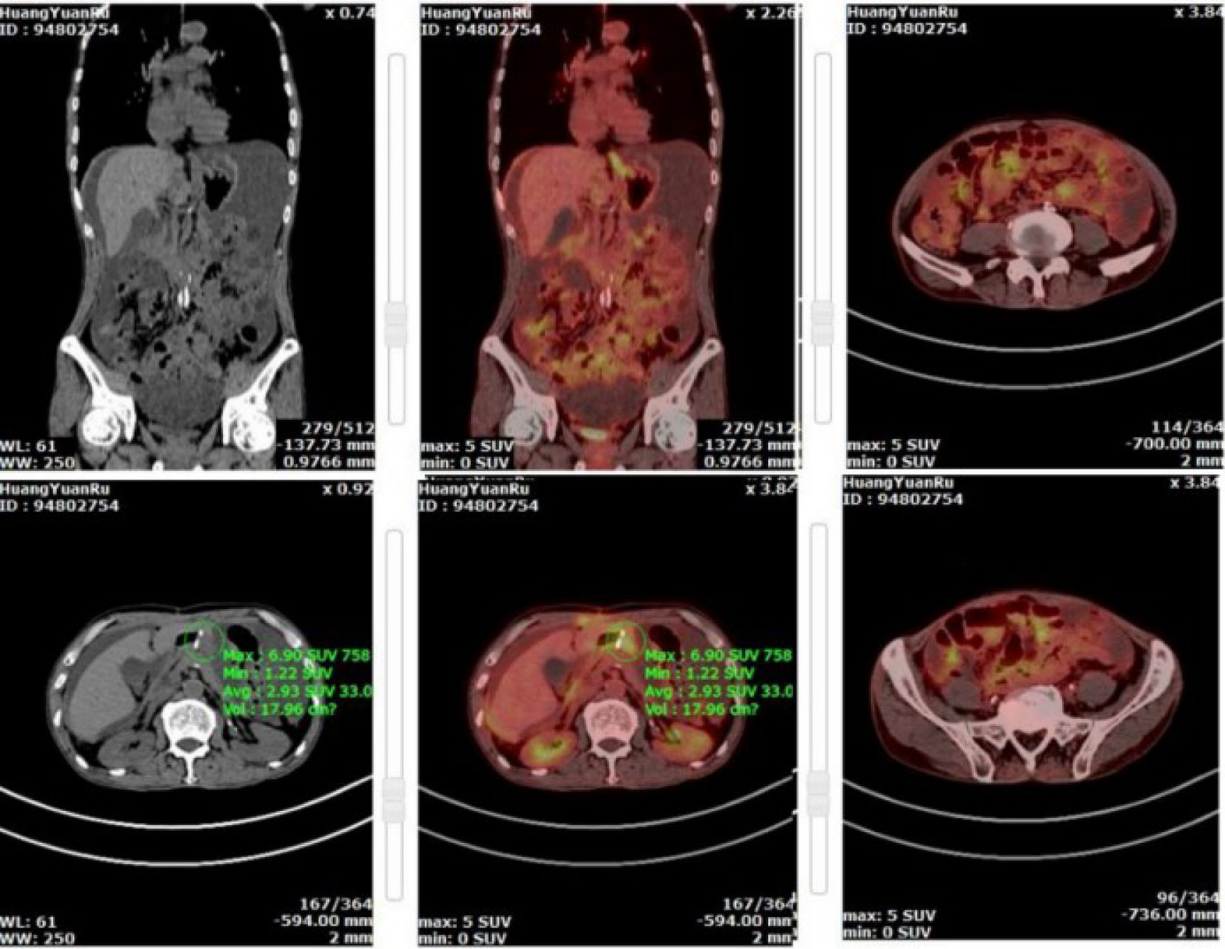
Pet-CT showed tumor recurrence at anastomosis with multiple peritoneal metastases.

**Figure 5:**
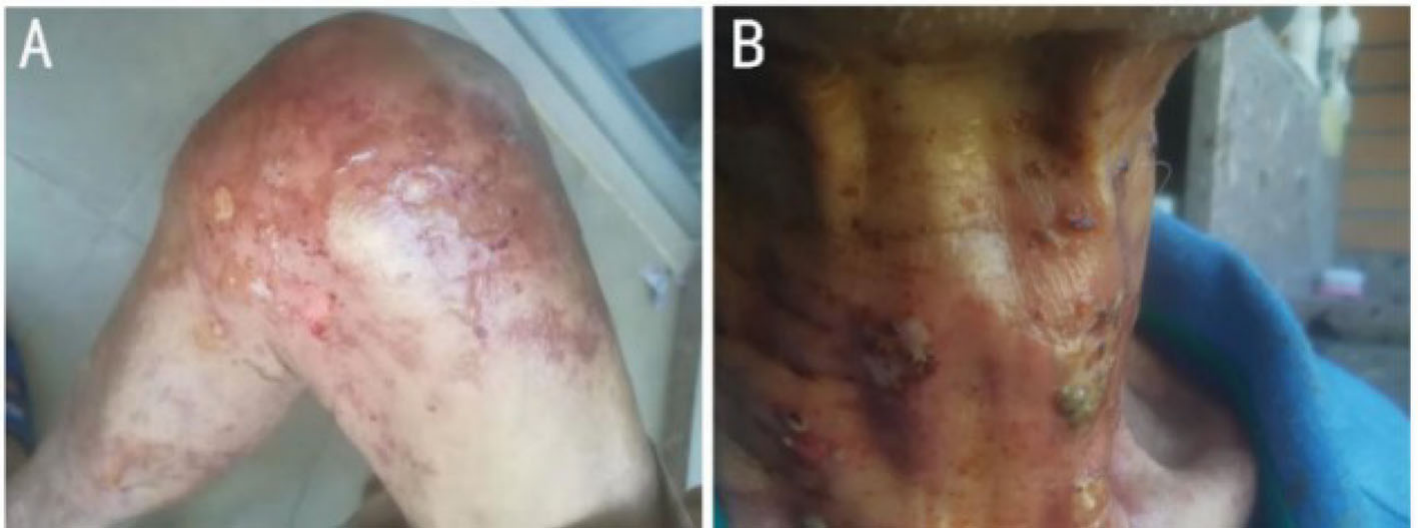
After the second cycle of combinative treatment, systemic inflammatory symptoms worsened and blisters appeared: **(A)**, lower limb rash and blister; and **(B)**, aggravation of neck rash and blister.

**Figure 6:**
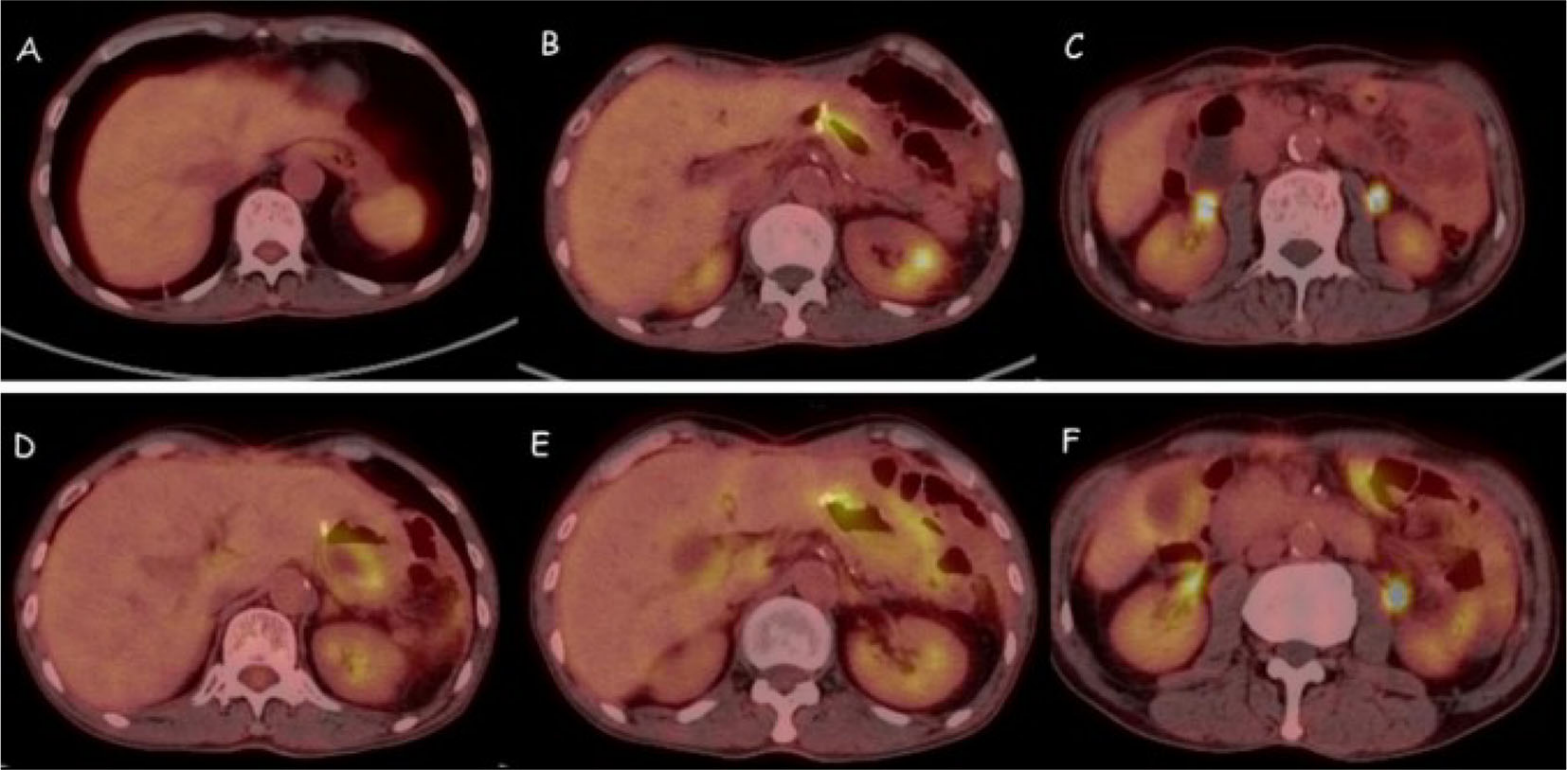
Pet-CT scan of peritoneal cavity after combinative treatment. **(A-C)** After 5 cycles of combined therapy, the abdominal metastases disappeared. **(D-F)** After 10 cycles of maintenance therapy, no significant recurrence and metastasis were observed.

A 68-year-old male patient was diagnosed with gastric antral adenocarcinoma (cT4aN2M0-1) by abdominal CT, pelvic CT, and gastroscopy in a local hospital. In February 2019, the patient received palliative distal gastrectomy in a local hospital. Postoperative pathological analysis showed a diagnosis of moderately differentiated adenocarcinoma, consistent with the preoperative diagnosis, and pathological staging was pT4bN3bM1, IVA. Postoperative chemotherapy was administered by using XELOX regimen. After discharge, the patient began to have symptoms of nocturnal back pain, frequent vomiting, and long and irregular defecation time. The patient came to our hospital on July 8, 2019. PET-CT scan in our hospital showed that the tumor recurred at the anastomotic site with multiple peritoneal metastases (**Figure 4**). After MDT discussion, a combined treatment regimen was performed on 2019/7/10. In particular, apatinib (500 mg/d) was administered orally for 10 days, followed by camrelizumab (200 mg, ivgtt, day 1, 14 days/cycle) and TAS-102 (20 mg, oral, days 1–21, 28 days/cycle). Apatinib (500 mg/d) was maintained during treatment. After the second cycle of combined treatment, the patient had inflammatory side effects such as rash and blister (**Figure 5**). By November 2019, we had completed five courses of treatment. At this time, PET-CT showed that the abdominal metastasis disappeared (**Figures 6A–C**). After the beginning of each cycle of treatment, C-reactive protein (CRP) increased significantly and returned to normal after the end of this combined treatment, accompanied by a rapid decrease in CA125 (**Table 1**). Considering the patient’s tolerance and the cumulative effect of chemotherapy-drug toxicity, the follow-up protocol was changed to camrelizumab (200 mg, ivgtt, day 1, 14 days/cycle) plus apatinib (500mg/d, oral). Ten cycles of maintenance therapy were administered from November 2019 to May 2020. PET-CT was performed on April 19, 2020 (**Figures 6D–F**), and no obvious recurrence or metastasis was found.

**Table 1:** Changes in CRP and CA125 before and after five treatment cycles.

Parameter	Time	First cycle	Second cycle	Third cycle	Fourth cycle	Fifth cycle
CRP (ng/ml)	1 day before treatment	2.3	4.5	1.2	2.3	1.2
	2 days after treatment	36.3	45.1	3.3	72.8	27.3
CA125 (ng/ml)	1 day before treatment	374.2	211.6	138.5	64.1	23.8
	End of this cycle	199.9	127.7	91.4	22.4	17.7

### 2.3 Patient 3

**Figure 7:**
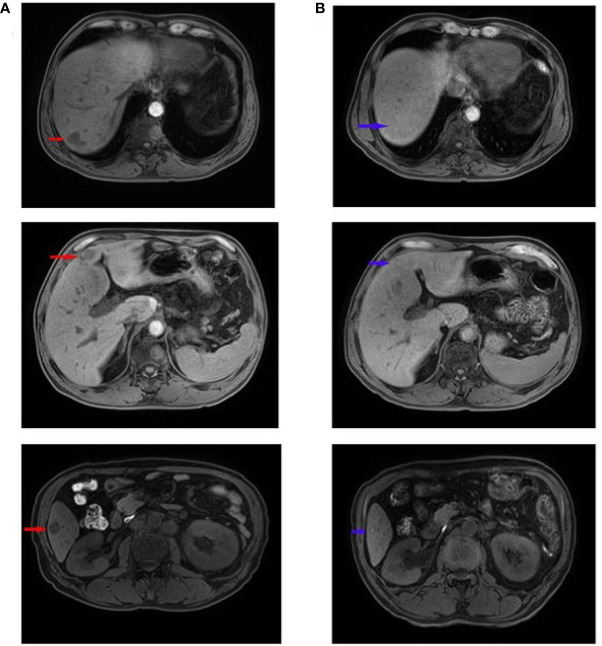
MRI images before and after combinative treatment. **(A)** Liver MRI metastases before the treatment of apatinib combined with camrelizumab and TAS-102. The larger one (with a size of about 2.5×1.7 cm) was located in the left medial lobe. The red arrow indicates the primary liver metastases. **(B)** Liver MRI metastases after the comprehensive treatment of apatinib combined with camrelizumab and TAS-102. The larger one (a diameter of about 1.0 cm) was located in the posterior upper segment of the right lobe of liver. The blue arrow indicates that the liver metastases shrunk or disappeared.

**Figure 8:**
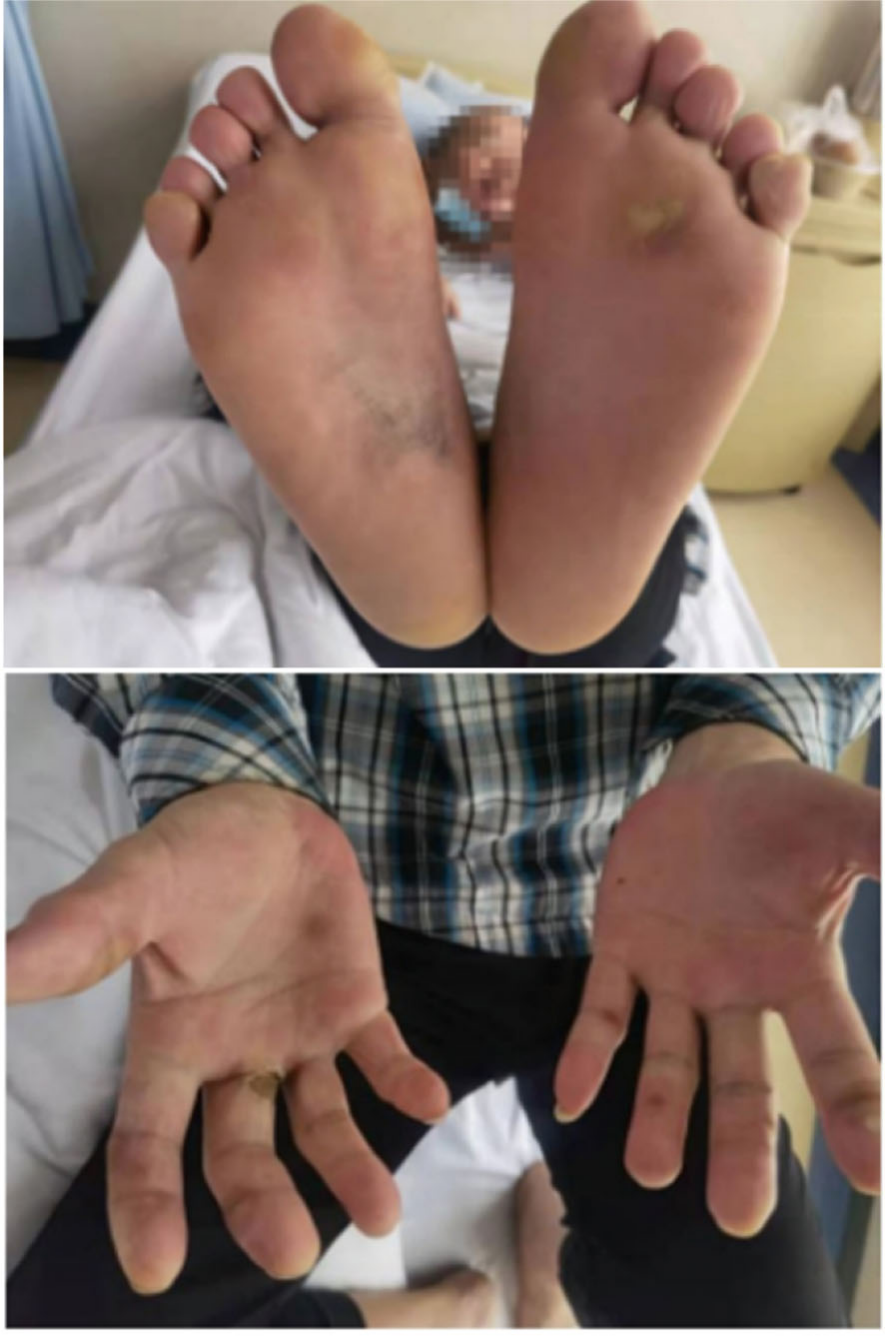
Development of rash on hands and feet during medication on the third day after the use of camrelizumab.

**Figure 9:**
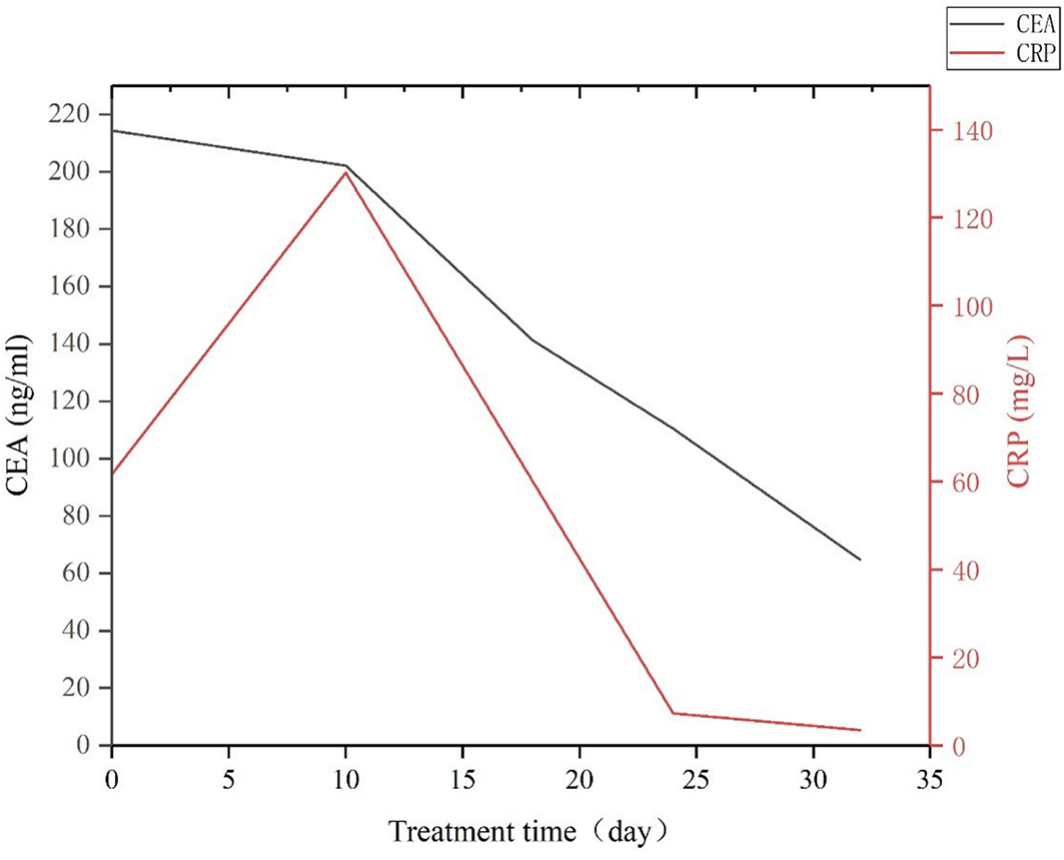
CEA and CRP expression levels changed during the early stage of treatment using apatinib combined with camrelizumab and TAS-102.

**Figure 10:**
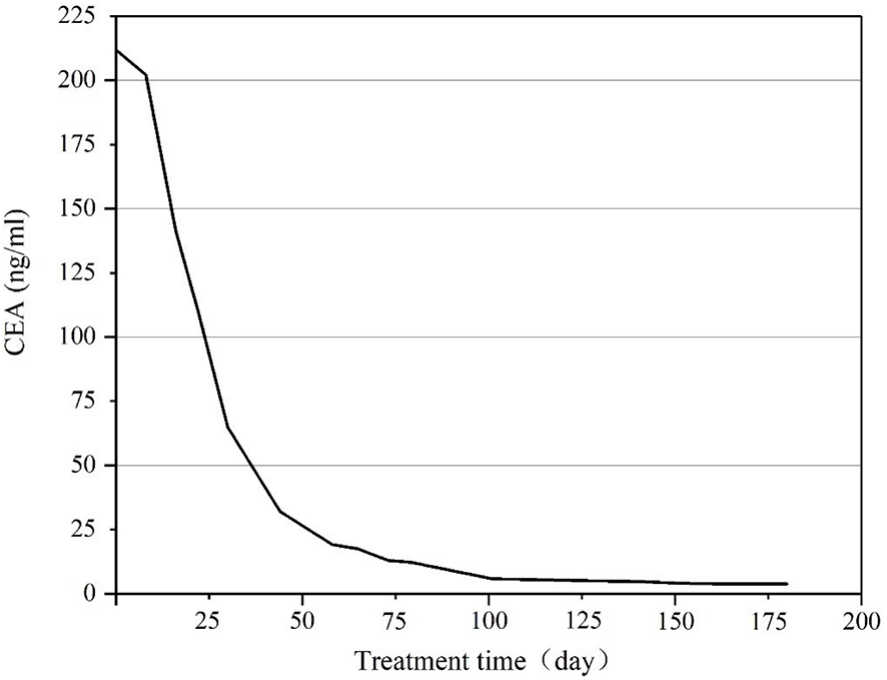
CEA level during treatment using apatinib combined with camrelizumab and TAS-102.

A 56-year-old male patient was diagnosed with moderately differentiated adenocarcinoma of the sigmoid colon in October 2019. On October 16th, 2019, the patient received a laparoscopic radical resection of sigmoid colon cancer. Postoperative pathological analysis showed a diagnosis of ulcerative moderately differentiated adenocarcinoma, consistent with the preoperative diagnosis, and the pathological staging was pT4N2aM0, IIIC. On November 25th, 2019, the patient was treated with Capeox regimen. In particular, oxaliplatin (130 mg/m^2^ of body surface area, ivgtt once a day on day 1) and capecitabine (875 mg/m^2^ of body surface area, orally twice a day on days 1–14, 21 days/cycle) were administered. On June 18th, 2020, the patient was re-examined by PET-CT, revealing multiple metastases of the liver and retroperitoneal lymph node. This indicated that the first-line chemotherapy regimen had failed. On July 17th, 2020, we changed the medicament regimens to the following: cetuximab (dosage 400 mg/m^2^ of body surface area, ivgtt for 120 min in first cycle, and then decreased to 250 mg/m^2^ of body surface area, ivgtt for 60 min; 21 days/cycle) plus FOLFIRI (irinotecan 180 mg/m^2^ of body surface area plus calcium folinate 400 mg/m^2^ of body surface area, ivgtt once a day on days 1–2, 14 days/cycle; 5-FU 400 mg/m^2^ of body surface area, iv in first cycle, and then 1200 mg/m^2^ of body surface area, ivgtt over 22 h, 14 days/cycle). On February 22nd, 2021, MRI re-examination showed tumor progression again (**Figure 7A**). After MDT discussion, a combined treatment regimen was performed on 2021/2/24. In particular, apatinib (500 mg/d) was administered orally for 10 days, followed by camrelizumab (200 mg, ivgtt, day 1, 14 days/cycle) and TAS-102 (20 mg, oral, days 1–21, 28 days/cycle). Apatinib (500 mg/d) was maintained during treatment. On the third day after the use of camrelizumab, the patient developed a marked skin rash (**Figure 8**) accompanied by oral ulcers, hoarseness, and elevated CRP. In the course of CRP elevation, the CEA level of the patient decreased (**Figure 9**). After four cycles of the protocol, the CEA level decreased significantly (**Figure 10**). On May 8th, 2021, MRI re-examination showed that the multiple metastatic tumors in the right lobe and left medial lobe of the liver and the retroperitoneal and para-aortic lymph nodes significantly shrunk compared with the last MRI (**Figure 7B**).

## 3 Discussion

The treatment of MSS/mismatch repair proficient (pMMR) advanced GI tumors remains under exploration. The feature of this series of cases was that after the failure of the original chemotherapy regimen, the patients with MSS/pMMR advanced GI tumors were treated using apatinib combined with camrelizumab, which induced a systemic inflammatory response (characterized by sharply increased CRP and emergence of systemic rash, oral ulcer, and gingivitis), and further combination with chemotherapy achieved good results. Patients 1 and 3 achieved partial response, and patient 2 achieved complete response. The reason for the good therapeutic effect may be related to the mechanism of targeted therapy combined with immunotherapy and chemotherapy. Moreover, we suspect that it may also be related to the inflammatory response caused by combined treatment.

### 3.1 Treatment plan for this series of cases

Apatinib is an oral small-molecule anti-angiogenesis drug that can selectively inhibit vascular endothelial growth factor receptor (VEGFR)-2 and slightly inhibit the activities of c-kit proto-oncogene protein and c-src tyrosine kinase (17). Apatinib can inhibit the VEGFR2/STAT3/Bcl-2 signaling pathway and increase the expression of beclin-1 *in vivo*, thereby inducing autophagy and apoptosis of tumor cells (18). Additionally, the administration of apatinib at low doses can alleviate hypoxia, increase the infiltration of CD8^+^ T cells, reduce the recruitment of tumor-associated macrophages, and lower the level of transforming growth factor-β in both tumor and serum, thereby changing the tumor microenvironment (TME) and enhancing the activity of anticancer drugs (19). In 2014, the drug was approved and listed in China to treat patients with advanced gastric adenocarcinoma or gastric esophageal junction adenocarcinoma who had progressed or relapsed after receiving at least two kinds of systematic chemotherapy. Currently, phase II/III clinical trials are being conducted in China, and good clinical results have been achieved in the treatment of GI tumors (20–23).

Camrelizumab is a humanized anti-PD-1 IgG4 monoclonal antibody also known as SHR-1210. Camrelizumab can bind to PD-1 and block the interaction with PD-L1 to prevent the activation of PD-1 and its downstream signal pathway, and restore immune function by activating the immune response against tumor cells or pathogens mediated by cytotoxic T lymphocytes (CTLs) (24). In 2019, camrelizumab was approved by the State Drug Administration of China for the treatment of recurrent or refractory classical Hodgkin’s lymphoma (24). Currently, many studies in China have reported its clinical potential in the treatment of different solid tumors (25–27).

TAS-102 is an oral combination drug composed of thymidine analog trifluridine (FTD) and a new thymine phosphorylase inhibitor tipiracil hydrochloride (TPI). FTD can replace thymine in the DNA chain, causing DNA function damage and playing an antitumor role. FTD also can inhibit the activity of thymidylate synthase, thereby blocking the pathway of thymidine synthesis from uracil. TPI can improve the bioavailability of FTD and prolong the half-life of FTD by inhibiting the activity of thymidine phosphorylase. TPI can also inhibit the neovascularization induced by platelet-derived endothelial cell growth factor/thymidine phosphorylase (28, 29). In multiple clinical trials, TAS-102 significantly improved the OS and progression-free survival (PFS) of patients with advanced or metastatic GI tumors compared with the placebo group (30–33).

### 3.2 Tumor microenvironment

TME is a complex environment in which tumor cells survive and develop. It primarily comprises immune cells and their secretory factors, vascular endothelial cells, mesenchymal-derived cells, extracellular matrix, and many others (34). The development of scientific technology has enabled different types of cells to be identified in the microenvironment, such as stromal cells, fibroblasts, fat cells, vascular endothelial cells, and immune cells (e.g., T lymphocytes, B lymphocytes, natural killer (NK) cells, tumor-associated macrophages, and so on) (35). Mesenchymal cells and fibroblasts can secrete fibroblast growth factor and vascular endothelial growth factor (VEGF) in the TME to promote the growth, invasion, and metastasis of malignant tumor cells (36). Vascular endothelial cells provide oxygen to tumor cells and synergistically promote tumor growth by inducing the formation of new blood vessels along with VEGF (37). Adipose tissue can induce tumorigenesis, progression, and metastasis by releasing pro-inflammatory factors and extracellular vesicles (38). Immune cells are the most important defense weapons of the human body, and they can resist the invasion or infection of harmful pathogens and eliminate damaged or cancerous cells. Immune cells in TME include T cells, Treg cells, NK cells, dendritic cells (DCs), myeloid-derived suppressor cells (MDSCs), macrophages, and so on (39). Tumor-infiltrating T cells are important effector cells in the immune system, and they can be categorized into helper T cells (CD4^+^ T cells), cytotoxic T cells (CD8^+^ T cells) and regulatory T cells (Tregs). CD8^+^ T cells can secrete interferon (INF)-γ, tumor necrosis factor (TNF)-α, and other antitumor factors to induce apoptosis (40). CD4^+^ T cells can differentiate into many types of immune cells and play different roles in immune response (41). Tregs and regulatory B cells are immunosuppressive cells in the immune system that inhibit the immune response of T lymphocytes to prevent the damage caused by the excessive activation of T cells (42, 43). The main function of NK cells is to exert cytotoxicity, and INF-γ, TNF-α, and granulocyte-macrophage colony-stimulating factor can be secreted after activation to exert an antitumor effect (44). DCs can express co-stimulating molecule and innate inflammatory cytokine to promote Th1 and CTL responses (45). MDSCs are also a type of immunosuppressive cells that can inhibit the activity of cytotoxic T cells by producing arginase 1, upregulating nitric oxide synthase, and reactive oxygen species (46). Neutrophils are distributed mostly in the peripheral blood, promoting the growth and metastasis of tumor cells by producing large amounts of proteases and growth factors (47). Macrophages can be divided into M1 and M2 macrophages. M1 macrophages have antitumor properties, which can upregulate pro-inflammatory cytokines such as interleukin (IL)-1b, IL-12, and TNF-α, and present antigens through major histocompatibility complex class II molecules. M2 macrophages have tumor-promoting properties, which can secrete anti-inflammatory factors such as IL-10, reduce the expression of pro-inflammatory factors, and inhibit adaptive immune response (48). Extracellular matrix is composed of basement membrane and intercellular matrix. It contains a large number of growth factors, laminin, acidic substances, collagen, and other components. These substances promote tumor growth and metastasis by participating in angiogenesis, selectively passing through small-molecule substances and promoting matrix sclerosis (49).

### 3.3 Tumor blood vessels

Tumor blood vessel is an important part of the TME. Its formation is a crucial step for tumor cell growth, proliferation, local invasion, and metastasis (50). During the process of tumor growth, the overexpression of angiogenic factors leads to the formation of these pathological blood vessels, which provide necessary nutrition for proliferating cancer cells (51). VEGFRs are tyrosine kinase receptors that are overexpressed in most solid tumors and they are commonly considered to be the key factor affecting tumor angiogenesis (52, 53). The VEGFR family of proteins mostly comprises three subtypes, namely VEGFR-1 (FMS-like tyrosine kinase-1), VEGFR-2 (kinase insert domain protein receptor 2), and VEGFR-3 (FMS-like tyrosine kinase-4). Among them, VEGFR-2 is the primary mediator of VEGF-induced angiogenesis signaling (54). A study has shown that the TME, characterized by hypoxia, low pH, and high interstitial fluid pressure, can reduce the effectiveness of almost all types of anticancer treatments (55). Therefore, normalizing a certain indicator of TME may improve the effectiveness of various treatments (56). Anti-VEGF therapy could prune immature blood vessels with low pericyte coverage, and normalize blood vessels through active pericyte supplementation (57, 58). Similar changes in vascular normalization were also observed in glioblastoma patients treated with VEGFR tyrosine kinase inhibitors (59, 60). Thus it can be seen that the role of vascular-targeted drugs in inducing vascularization around tumors has become a breakthrough in changing the TME. Ramjiawan et al. (61) believe that a reasonable dose of anti-angiogenesis drugs can temporarily normalize the tumor vascular system by reducing vascular permeability and improving tumor blood perfusion. When combined with immunotherapy during the window of normalization, a more favorable treatment outcome can be attained. Accordingly, during the window of vascular normalization, the administration of immune drugs and chemotherapy drugs has become a key point for the success of combination therapy. However, one of the existing challenges is that the duration of vascular normalization is brief and varies based on the tumor type and the dosage of antiangiogenic medication administered (60, 62). In this regard, we observed a special phenomenon during the treatment of three patients: all three patients showed inflammatory reactions such as a surge in CRP, rash, and oral ulcer at different stages after combined treatment. One study showed that pre-administration of anti-VEGF drugs before the induction of colitis in mice could exacerbate the inflammatory response and significantly reduce the vascular density in the colon at the end of the acute phase of inflammation (63). Thus, we speculate that the occurrence of a systemic acute inflammatory response may mean that blood vessels begin to be in a window of normalization, and that the timing of drug administration at the transition point between acute and non-acute phases of the inflammatory response may be more effective.

### 3.4 Anti-angiogenesis therapy combined with chemotherapy

Tumor blood vessels are generally hyperpermeable, leading to the inability of tumor vessels to maintain the gradient of vascular and interstitial pressure. This lack of pressure gradient also impairs the flow of fluid and macromolecules (64). Therefore, normalizing blood vessels through anti-angiogenesis drugs can provide a crucial pathway for anticancer drugs to enter the tumor. This has been confirmed by Tsukihara et al. (65). Their study showed that the combination of bevacizumab and TAS-102 treatment could increase the accumulation of FTD in tumors and further promote its phosphorylation, compared with TAS-102 monotherapy. In addition, in the process of chemotherapy, tumor cells develop multidrug resistance (MDR) by increasing drug efflux, reducing drug uptake, target mutation or other methods (66). The most common cause of MDR is the overexpression of ATP-binding cassette transporter bound to cell membrane, which actively pumps multiple chemotherapeutic drugs out of cancer cells, thereby reducing their cytotoxicity (67). However, apatinib could reverse the MDR of tumor by inhibiting the efflux function of ATP-binding cassette transporters (68). Currently, clinical trials on anti-angiogenesis drugs combined with chemotherapy for the treatment of GI tumors have achieved promising results. In a phase II clinical trial, 90 patients were randomized to combination group (sunitinib plus FOLFIRI) or single-drug group (sunitinib plus placebo), with 45 people in each group. The result showed that the median OS in the combination group was significantly longer than that in the single-drug group (10.4 months vs. 8.9 months) (69). In a phase III clinical trial reported by Wilke et al. (70), 330 patients received ramucirumab plus paclitaxel (combination group) and 335 patients received placebo plus paclitaxel (single-drug group). The median OS of the combination group was significantly longer than that of the single-drug group (9.6 months vs. 7.4 months). The proportion of patients who achieved objective response (OR) in the combination group was significantly higher than that in the single-drug group [92/330 (28%) vs. 54/335 (16%)]. The proportion of patients who achieved disease control in the combination group was significantly higher than that in the single-drug group [264/330 (80%) vs. 213/335 (64%)]. These results show that antiangiogenic therapy in combination with chemotherapy can have a better therapeutic effect.

### 3.5 Immunotherapy combined with chemotherapy

The efficacy of chemotherapy is attributed to its cytotoxic effects and the activation of immune surveillance, which promotes the development of an immunogenic environment within the tumor and stimulates the cancer-specific immune response (71). Immunogenic cell death is a form of cell death induced by radiotherapy, photodynamic therapy, or some cell inhibitors. When tumor cells undergo immunogenic cell death, they release a series of signal molecules called damage-associated molecular patterns (72), primarily including calreticulin on the cell surface, high mobility group protein 1secreted by tumor cells, ATP released by cells, and heat shock proteins (HSP70 and HSP90) (73). The exposure of calreticulin on the cell surface can stimulate DCs to phagocytize tumor cells (74). High mobility group protein 1 can induce the recruitment of CD8+t cells into TME (75). HSPs activate tumor cells to produce chemokines through the toll-like receptor (TLR)-4 signaling pathway, which attracts DCs and T lymphocytes (76). Damage-associated molecular patterns can activate DCs through TLR4 and enhance the induction of antitumor T-cell immune responses (77). The increased concentration of extracellular ATP can recruit DCs and T cells into the tumor (78). Chemotherapeutic drugs can also provide a favorable antitumor immune microenvironment by directly eliminating Tregs, MDSCs, or M2 macrophages. One study showed that mice with multiple injections of gemcitabine had a decreased percentage of MDSCs and Tregs in their spleen and tumor tissue compared to controls treated with phosphate-buffered saline (79). Additionally, low-dose cyclophosphamide could deplete Tregs and inhibit its immunosuppressive activity, as well as inhibit the polarization of M2 macrophages, thereby interfering with the formation of inhibitory immune microenvironment (80, 81). Other chemotherapeutic drugs such as adriamycin, cisplatin, or paclitaxel could increase the permeability of intracellular granzyme B, rendering tumors more susceptible to the cytotoxicity of CTLs (82). In a phase II trial, patients with CRC were administered subcutaneous injection of granulocyte-macrophage colony-stimulating factor and low-dose IL-2 following chemotherapy with gemcitabine plus FOLFOX-4 (oxaliplatin, fluorouracil, and folic acid). Following a median follow-up period of 12.5 months, the objective response rate (ORR) and disease control rate was as high as 68.9% and 96.5%, respectively. Detection results of peripheral blood mononuclear cells in 20 patients showed that the antigenic immune response of colon cancer was enhanced, and the inhibitory regulatory T lymphocytes (CD4^+^ CD25T-reg^+^) were significantly reduced (83). These results indicate that the combination of chemotherapy and immunotherapy can effectively enhance the immune response and improve treatment outcomes.

### 3.6 Mechanism of combined application of anti-angiogenesis therapy and immunotherapy

Targeted therapies based on anti-angiogenesis drugs are increasingly applied to the clinical treatment of various tumors. The mechanism of immunotherapy combined with anti-angiogenesis therapy is that it can inhibit angiogenesis and reprogram TME (16). As early as 2013, Yasuda et al. (84) observed in colon adenocarcinoma mice that the simultaneous use of anti-PD-1 and anti-VEGFR2 monoclonal antibodies could synergistically inhibit tumor growth *in vivo*. Voron et al. (85) found that VEGF-A produced in the TME could enhance the expression of PD-1 and other inhibitory checkpoints involved in CD8^+^ T cell failure, which may be reversed by relevant vascular-targeted drugs. This finding has been confirmed by Meder et al. (86). Through a mouse model of small-cell lung cancer, they discovered that mice with resistance to PD-L1 treatment showed a significant increase in the expression of exhausted T cells. However, after undergoing anti-VEGF combined with anti-PD-L1 treatment, the increase in the proportion of exhausted T cells could be reversed. This shows the efficacy of anti-PD-1 combined with anti-VEGF therapy in effectively blocking the PD-1/PD-L1 axis and synergistically suppressing tumor growth. Additionally, the combination therapy of anti-VEGFR-2 and anti-PD-L1 could also improve anti-PD-L1 therapy by inducing the production of high endothelial veins within tumors, promoting the infiltration of CTLs around high endothelial veins, and enhancing the activity of CTL (87).

### 3.7 Current status of anti-VEGF/VEGFR plus anti-PD-1 combined with/without chemotherapy in the treatment of MSS gastrointestinal tumors

Preclinical studies have shown the gain effect of vascular-targeted therapy combined with immunotherapy, but the effect on patients with MSS GI tumors is unsatisfactory. Eng et al. (88)reported a phase III study, in which 363 patients with MSS metastatic colorectal cancer (mCRC) were treated with atezolizumab plus cobimetinib or atezolizumab monotherapy or regorafenib monotherapy. Following a median follow-up period of 7.3 months, the median OS, PFS, and ORR of atezolizumab combined with cobimetinib group was 8.87 months, 1.19 months, and 3% respectively. The median OS, PFS, and ORR of the atezolizumab group was 7.10 months, 1.94 months, and 2% respectively. The median OS, PFS, and ORR of the regorafenib group was 8.51 months, 2 months, and 2% respectively. Overall, no significant differences exist in median OS, PFS, and ORR among the three groups. Cousin et al. (89) reported a clinical trial that among 48 patients with MSS mCRC who received Regorafenib plus Avelumab combination therapy, none of the patients achieved OR. Similarly, in a phase II study, 10 patients with MSS mCRC received shr-1210 (anti-PD-1) combined with apatinib, and none of patients (0%) achieved OR, and only 2 patients (22.2%) achieved disease control (90). Although the above studies suggest that patients with MSS GI tumors may not respond well to anti-angiogenesis therapy combined with immunotherapy, there are other studies that have shown good results. In a phase II clinical trial involving 50 patients (25 with GC and 25 with CRC) treated with regorafenib, a total of 20 patients (40%) achieved OR, including 11 patients with GC (44%) and 9 patients with CRC (36%) (91). Gomez Roca et al. (92) also reported promising results from a phase II clinical trial that 32 patients with MSS CRC treated with lenvatinib plus pembrolizumab and an ORR of 22% was observed. Additionally, in a phase IB clinical study, vascular-targeted therapy combined with immunotherapy also showed exciting antitumor activity. Following the administration of regorafenib in combination with nivolumab, the ORR achieved 36% (9/25) in 25 patients with MSS mCRC (93). Other clinical trials also showed different clinical effects (**Table 2**). The inconsistent outcomes indicate that the efficacy of anti-angiogenesis therapy combined with immunotherapy for patients with MSS GI tumors is still debatable, which may be related to whether the drug is administered at the time point of vascular normalization.

**Table 2:** Summary of clinical trials investigating the use of immunotherapy-based combinations for MSS gastroenteric tumor.

Study	Treatment	Phase	Sample Size	ORR	Median PFS	Median OS
Immunotherapy in combination with chemotherapy and anti-VEGF agents						
Grothey, A (94)	FOLFOX + bevacizumab followed by 5-FU + bevacizumab+ atezolizumab vs. 5-FU + bevacizumab	II	297 vs. 148	NR	7.2 vs. 7.39 months	22 vs. 21.9 months
Cremolini, C (95)	FOLFOX + bevacizumab + atezolizumab vs. FOLFOX + atezolizumab	III	132 vs. 67	59% vs. 64%	12.9 vs. 11.4 months	NR
Mettu, N (96)	Capecitabine/bevacizumab + atezolizumab vs. capecitabine/bevacizumab + placebo	II	82 vs. 46	8.5% VS 4.4%	4.4 vs. 3.6 months	10.3 vs. 10.2 months
Antoniotti, C (97)	FOLFOX + Bevacizumab + Atezolizumab vs. FOLFOX + Atezolizumab	II	145 vs 73	NR	13.0 vs 11.0 months	NR
Immunotherapy in combination with antiangiogenic agents						
Gomez-Roca (92)	Pembrolizumab + lenvatinib	II	32	22%	2.3 months	7.5 months
Kim, R (93)	Nivolumab + regorafenib	Ib	25	36%	7.9 months	NR
Cousin, S (89)	Avelumab + regorafenib	II	48	0%	3.6 months	10.8 months
Cathy Eng (88)	Atezolizumab + cobimetinib vs. atezolizumab vs. regorafenib	III	183 vs. 90 vs. 90	3% vs. 2% vs. 2%	1.19 vs. 1.94 vs. 2 months	8.87 vs. 7.10 vs. 8.15 months
Ren, C (90)	SHR-1210+apatinib	II	10	0%	1.83 months	7.80 months
Li, J (98)	Regorafenib + anti-PD-1 antibody	NR	23	NR	3.1 months	NR
Fukuoka, S (91)	Regorafenib + nivolumab	IB	50	40%	5.6 months gastric cancer 7.9 months colorectal cancer	12.3 vs. NR months
Martinelli, E (99)	Cetuximab + avelumab	II	77	NR	3.6 months	11.6 months
Wang, F (100)	Regorafenib + toripalimab	Ib/II	33	15.2%	2.1 months	15.5 months
Zhang,W (101)	Fruquintinib + sintilimab	II	55	16%	4.1 months	13.3 months

### 3.8 Acute inflammation with tumors

Chronic inflammation promotes the growth and invasion of cancer through factors produced by immune cells (such as cytokines, growth factors, and reactive oxygen species), which has become a basic consensus (102). However, the effects of acute inflammation on tumors have not been extensively studied. Acute inflammation is a complex process that is responsible for controlling tissue damage caused by pathogenic, traumatic, or toxic injury. This process is closely coordinated by pro-inflammatory and anti-inflammatory molecules that regulate chemotaxis, migration, and cell activation. It is characterized by the rapid recruitment and activation of white blood cells (such as neutrophils, eosinophils, and NK cells) which infiltrate the inflamed area to remove the remaining pathogens (103). Hobson et al. (104) found that breast biopsies could induce the aggregation of inflammatory cells at the biopsy site of mice with breast cancer, and the proportion of tumor cell proliferation in the surrounding area of the biopsy site significantly increased. Furthermore, the mice that underwent biopsies also showed a significant increase in the number of new lung metastases. Another study also demonstrated the promoting effect of acute inflammation on tumor development. In pancreatic cancer mice with more severe acute inflammatory response, the expression of snail protein, epithelial cell adhesion molecule, mucin 1, NOD-like receptor protein 3 and microRNA-155 in the liver and pancreas significantly increased, and exhibited larger tumor volumes and higher liver metastasis rates (105). However, Ma et al. (106) observed in a mouse model of melanoma that as the degree of acute inflammatory reaction intensified, the tumor volume gradually decreased. Additionally, during the inflammatory process, high levels of INF-r expression were detected in both serum and tumors of the melanoma mice. Salem et al. (107) used TLR ligand to induce acute inflammation in ascites cancer mice, and found that the decrease in the number of Ehrlich ascites tumor cells is associated with the increased infiltration of inflammatory cells expressing the myeloid markers CD11b + ly6g +, CD11b + ly6g -, and CD11b-ly6g + in the tumor. This finding suggests that providing some inflammatory stimuli in the early or late stages of tumor progression can effectively induce tumor regression, which may be mediated by inflammatory cells (such as bone-marrow-derived cells). The study also discovered that injection of polyinosinic acid-polycytidylic acid could increase the number of macrophages (CD11b+) by 8-fold during the course of an acute inflammatory response (107). Interestingly, Schmid et al. (108) discovered that high levels of CD11b expression could promote the polarization of inflammatory macrophages (M1 myeloid cells) in the TME and stimulate the accumulation of CD8^+^ T cells in tumors, thereby changing the TME and exerting an antitumor effect. Regarding the external manifestation of acute inflammation, such as rash, some observational studies involving antiangiogenic drugs found that patients who developed a rash during treatment had longer survival time and better treatment efficacy compared with those who did not, but no clear explanation has been provided (109, 110). While some studies considered the increase of CRP before treatment as an indicator of poor prognosis (111), or thought that myeloid cell infiltration caused by the increase of CRP before operation counteracted the beneficial effects and potential tumor-suppressing effects of lymphocyte infiltration (112), which seem to be contradictory to the treatment results of our cases. Accordingly, we have made the following two assumptions. First, the factors causing the increase in CRP differed. In our series of cases, the sharp rise in CRP in cases 2 and 3 occurred after drug treatment, whereas the subjects in studies by Spencer et al. (111) and Kostner et al. (112)had not received any treatment, and the increase in CRP of their subjects may be related to tumor metabolism. Second, the difference in CRP increase during treatment may also affect the treatment effect. These possibilities require further confirmation through randomized controlled trials.

## 4 Conclusion

The relationship between tumors and the body can be likened to that of weeds and trees. In summer, weeds and trees mix together and are difficult to separate (immune tolerance). However, the change in human body environment is similar to cooling from summer to winter. In winter, both weeds and trees undergo a process of withering, however, the extent of withering is more severe in weeds than in trees. As a result, it becomes possible to clearly distinguish between the two (decreased immune tolerance). In this scenario, removing the weeds is more feasible. In other words, by creating an acute inflammatory environment in the body, we have enabled a specific separation between malignant tumors and the body. Before coming back in spring, drugs (sickles) can easily, quickly, and cleanly eliminate cancer cells (weeds). Our cases prove this hypothesis.

In the author’s recent clinical experience, more than 100 patients with advanced GI tumors received a combination of antiangiogenic therapy, immunotherapy, and chemotherapy. Among these patients, only eight developed an acute inflammatory reaction during a certain stage of treatment. However, after completing the treatment, all patients achieved satisfactory results in both imaging and serological manifestations. This report focuses on three typical cases where the use of apatinib and camrelizumab induced a systemic acute inflammatory response in patients with MSS advanced GI tumors, resulting in good outcomes when combined with chemotherapy.

Therefore, inducing systemic acute inflammatory response through the combination of vascular targeted drugs and PD-1/L1 monoclonal antibodies, followed by the use of chemotherapy, may lead to unexpected favorable outcomes. The therapeutic regimen may be a breakthrough strategy in the comprehensive treatment of tumors in the future, offering hope for the treatment of malignant tumor patients with failed treatment of multiple metastases. Nevertheless, further randomized controlled trials are still required to confirm these findings.

## Author contributions

SQM and LGY designed the “ideas”; CSY helped LYH and LL to deal with the information efficiently; LYH, LL, and CSY wrote the manuscript; LGY, DCC, ZRJ, CWP, and LQ revised the manuscript. All authors contributed to the article and approved the submitted version.
